# Induced Pluripotent Stem Cells for Treatment of Alzheimer’s and Parkinson’s Diseases

**DOI:** 10.3390/biomedicines10020208

**Published:** 2022-01-19

**Authors:** David A Yefroyev, Sha Jin

**Affiliations:** Department of Biomedical Engineering, Thomas J. Watson School of Engineering and Applied Sciences, State University of New York at Binghamton, Binghamton, NY 13902, USA; dyefroy1@binghamton.edu

**Keywords:** induced pluripotent stem cells, Alzheimer’s disease, Parkinson’s disease, neural cells, neural organoids, 3D bioprinting

## Abstract

Neurodegenerative diseases are a group of debilitating pathologies in which neuronal tissue dies due to the buildup of neurotoxic plaques, resulting in detrimental effects on cognitive ability, motor control, and everyday function. Stem cell technology offers promise in addressing this problem on multiple fronts, but the conventional sourcing of pluripotent stem cells involves harvesting from aborted embryonic tissue, which comes with strong ethical and practical concerns. The keystone discovery of induced pluripotent stem cell (iPSC) technology provides an alternative and endless source, circumventing the unfavorable issues with embryonic stem cells, and yielding fundamental advantages. This review highlights iPSC technology, the pathophysiology of two major neurodegenerative diseases, Alzheimer’s and Parkinson’s, and then illustrates current state-of-the-art approaches towards the treatment of the diseases using iPSCs. The technologies discussed in the review emphasize in vitro therapeutic neural cell and organoid development for disease treatment, pathological modeling of neurodegenerative diseases, and 3D bioprinting as it applies to both.

## 1. Introduction

**Figure 1:**
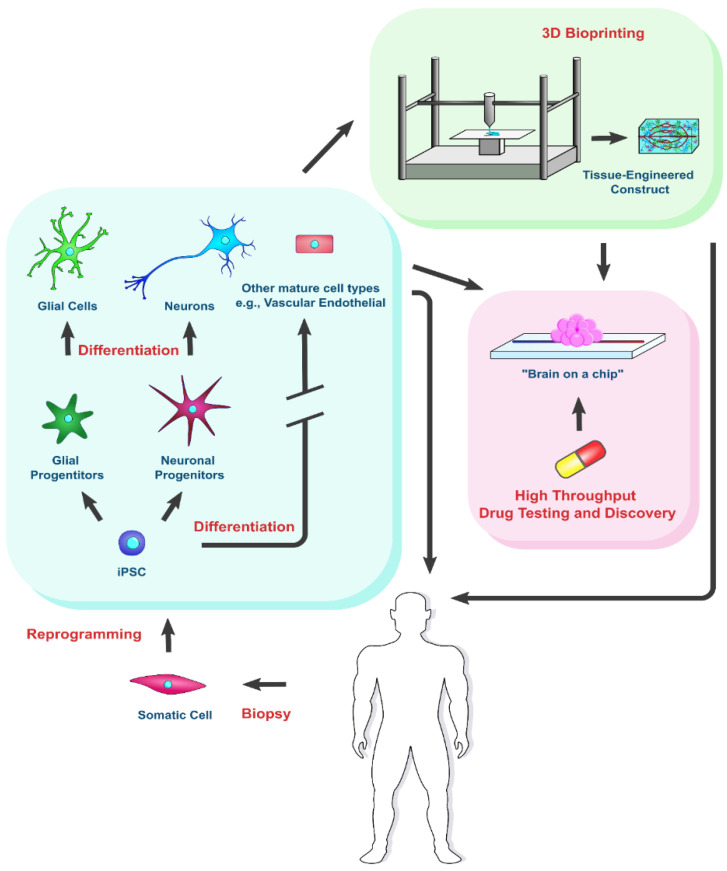
iPSCs for the treatment of neurodegenerative diseases. iPSCs are reprogrammed from the patient’s somatic cells. Their derived cells can be implanted directly or used to fabricate constructs for drug testing. Bioprinting can be applied to achieve the expected tissue structure of the engineered constructs.

Neurodegenerative diseases (ND), including Alzheimer’s disease (AD) and Parkinson’s disease (PD), afflict some 50 million people in the US alone, with over 600 types having been identified as of 2017 [1]. Currently, there is no cure for Parkinson’s nor Alzheimer’s diseases. Induced pluripotent stem cells (iPSCs) provide an endless cell source for the generation of functional human neural cells for cell-based therapy and for in vitro modeling. This review highlights iPSC technology, the pathophysiology of the two major neurodegenerative diseases, and current state-of-the-art approaches towards treatment of the diseases using iPSC-derived cells. Particularly, the review focuses on approaches for regenerative implantation and in vitro neural organoid development, in combination with the rapidly evolving field of 3D bioprinting, for potential neurodegenerative disease treatment and drug testing (Figure 1).

## 2. Alzheimer’s and Parkinson’s Diseases

Generally, AD occurs due to the cytotoxic buildup of protein aggregates, which are either misregulated or contain abnormal conformations [2]. Alzheimer’s disease is the leading type of neurodegenerative disease, accounting for 62% of the total cases [3]. Amyloid (Aβ) plaque accumulation is one of the key causes of neurodegeneration in AD, eliciting neuron apoptosis and necrosis. Its formation is regulated by beta and gamma secretase activity, which are more highly expressed and active in AD patients [4]. Yet another mechanism implicated in AD pathogenesis involves incorrect phosphorylation of Tau protein, leading to its aggregation and subsequent neurodegeneration [5].

Parkinson’s disease accounts for 2% of the total number of ND cases [6]. It is characterized by a loss of dopaminergic neurons in the substantia nigra, a nucleus acting as the primary input to the basal ganglia in the midbrain [7]. A parallel loss of other, non-dopaminergic neurons, also occurs, but there is a selective vulnerability of dopaminergic neurons, so they degrade at a faster rate [8]. Excessive alpha-synuclein accumulation is thought to be the main cause of PD, leading to the formation of clumps of protein or fibrillar aggregates, so-called Lewy bodies, in the brain. They play a deleterious role in microtubule function, resulting in neural degeneration. Overexpression of the gene coding for alpha-synuclein, or alterations of its amino acid sequence accelerate the protein aggregation [6]. In addition, environmental factors, such as toxic chemicals, are among the main factors contributing to the progression of Parkinson’s [1].

Although neurons are thought to be the main targets of regenerative cell therapy for ND, neuroglial cells, such as oligodendrocytes and astrocytes, play a pivotal role in ND pathology, and are therefore prospective targets for treatment and neuroprotection [9]. In nervous tissue, they regulate the biochemical environment, form the myelin sheath, and participate in the immune response. Hence, neuroglial cells support the development, and maintain the overall structural and functional integrity, of the nervous system ^9^. Dopaminergic neurons innervate spiny projection neurons (SPNs) in the striatal circuitry, performing an inhibitory role. SPNs are also innervated by glutaminergic neurons. A disbalance in the glutamate and dopamine regulation of SPN activation leads to glutamine spillover and subsequent excitotoxicity [10]. Mounting evidence has implicated glutamate dysregulation in the progression of both Parkinson’s and Alzheimer’s diseases [10,11,12]. Glial cells are crucial in the maintenance of the extracellular glutamate concentration, participating in reuptake and controlling glutamate spillover. A dyshomeostasis of glutamate leads to excitotoxicity of the region, as well as a neuroinflammatory cascade, further inhibiting the glial cells’ ability to regulate glutamate. Hence, regenerating damaged glial cells should be one of the directions for treatment of AD and PD.

It has recently been suggested that, owing to the difficulty in classifying neurodegenerative disease into discrete classes, it might be beneficial to model on a spectrum; each type is thought to be a combination of a number of factors. Some of these factors are plaque morphology, affected cell type, and the type of neural pathways affected [3]. Interestingly, it might also be the case that accumulation of plaque is not the original cause but rather the symptom, despite causing a further degradation of neural networks and glial cells [3].

## 3. Current Treatments for Alzheimer’s and Parkinson’s

Currently, there is no cure for Alzheimer’s nor for Parkinson’s diseases. The majority of current regimens are centered around alleviating/managing symptoms. Alzheimer’s is characterized by necrosis of cholinergic neurons. Thus, standard treatments take the form of cholinesterase inhibitors in order to compensate for decreased cholinergic neuron activity [13]. Memantine is usually taken in combination, which acts as an N-methyl-D-aspartate (NMDA) antagonist to counteract the glutaminergic excitotoxicity evident in AD progression [13]. Disease-modifying treatments are scarce, although a number of them are undergoing clinical trials. These include Tau aggregation inhibitors, monoclonal antibodies targeting amyloid plaque, and beta-secretase enzyme inhibitors [14]. The FDA just recently approved aducanumab, a monoclonal antibody treatment for AD, despite mixed results in early clinical trials [15].

For treating Parkinson’s, Levodopa (L-DOPA) along with a DOPA-decarboxylase inhibitor are commonly prescribed to patients [8]. L-DOPA is a precursor to dopamine that readily crosses the blood–brain barrier. It is, however, quickly taken out of the blood stream. Taking it with DOPA-decarboxylase inhibitors, such as carbidopa, gives it a maximum half-life of about 90 min [8,16]. Another treatment option involves directly taking dopaminergic agonists to supplement natural dopamine. Monoamine oxidase-B (MAO-B) and catechol-o-methyl transferase (COMT) inhibitors can also be taken to decrease dopamine catabolism, increasing overall dopamine levels. However, these treatments typically entrain side effects such as dyskinesia [8], and can wear off in between doses, resulting in “off times”. Although far from ideal, optimizing delivery methods and drug release profiles for individual patients may reduce the “off times’’ of these medications.

For both AD and PD, deep brain stimulation (DBS) has shown promising results as it aims to stimulate damaged neural circuits, naturally consolidating their endurance [8,16]. This is quite invasive however, as it involves implanting an electrode in specific brain regions. In Parkinson’s patients, it alleviates bradykinesia, dyskinesia, and tremor, although it has been known to exacerbate other symptoms in some cases [8].

## 4. Prospect of Human Pluripotent Stem Cells

Human pluripotent stem cells (hPSCs) are receiving much attention in the field of tissue regeneration. They can be differentiated into any cell type in the human body and subsequently developed into heterogeneous tissues or organs in vitro for implantation. Conventionally, hPSCs are harvested from surplus embryos that were generated after in vitro fertilization, known as embryonic stem cells (ESCs). A number of clinical trials employing human embryonic stem cell (hESC)-derived tissue have been performed. Many of them are focused on the treatment of macular degeneration, and have shown promise in improving visual acuity [17,18,19,20]. Likewise, a number of studies using ESCs were carried out in an attempt to treat ischemia [21] and even pulmonary fibrosis caused by COVID-19 [22]. Implantation of mesencephalic fetal tissue into the putamen has also exhibited promising results in the treatment of neurodegenerative diseases, particularly in younger patients [23]. Clinical trials have recently been completed for the treatment of Alzheimer’s using mesenchymal stem cell therapy [24]. The majority of these trials, however, required parallel immunosuppressive treatment, and obtaining these cells required harvesting them from a blastocyst, which presents an ethical issue: an embryo with a unique genome has to be destroyed, which would have otherwise developed into a human being [25]. If then used for regenerative implantation, fetal stem cells carry a high risk of invoking an immune rejection and graft-versus-host disease (GVHD) upon implantation, which is why immunosuppressive drugs would have to be taken postimplantation [26].

Other methods of obtaining pluripotent stem cells exist, such as somatic cell nuclear transfer (SCNT) [27] and induction of parthenogenesis [28]. The latter is a technique in which an unfertilized egg cell, or oocyte, is stimulated to divide. This can be undertaken at various points of meiosis. For example, a primary oocyte that still has a diploid nucleus may be stimulated to undergo mitosis. However, inherent problems with parthenogenesis, such as decreased protection against tumorigenicity owing to their homozygosity, as well as a lack of balanced epigenetic imprinting, are currently hurdles in its use for regenerative medicine [29]. SCNT, on the other hand, involves transferring a diploid nucleus from a differentiated somatic cell into an enucleated oocyte. The inefficiency and low availability of oocytes, however, hinder the prospect of using SCNT for regenerative medicine [30].

The groundbreaking discovery of iPSCs in 2006 by the Yamanaka group opened the door to a sustainable source of pluripotent stem cells, bypassing the ethical concerns and many of the technical hurdles associated with using conventional methods [31]. Using iPSCs offers an abundant source of stem cells that are autologous to a patient, removing the need for an immunosuppressive regimen postimplant, and allowing more accurate simulation of native tissue in vitro. Cells generated using this contemporary method would not be subject to immune rejection, as they are derived from the patient receiving the transplantation. This method revolves around the reprogramming of somatic cells, which, of course, has its own challenges, but nonetheless, remains a pivotal discovery for tissue engineering, and shows promise in the treatment of neurodegenerative disease. Remarkably, plenty of studies have demonstrated that iPSCs and ESCs are molecularly and functionally equivalent [32,33]. Importantly, iPSCs and ESCs exhibited similar lineage specification capability when they were compared side by side for the generation of a functional tissue or organ, including but not limited to neurons, pancreatic islet organoids, cardiomyocytes, and kidney micro-organoids [34,35,36,37,38,39,40,41]. This pivotal feature makes iPSCs valuable sources for biomedical applications. However, variations in differentiation exist among the different iPSC lines, due in part to the reprogramming methods applied during the creation of iPSC lines and the individual genetic features of donor cells [42,43]. Hence, iPSC line selection should be taken into account accordingly to develop strategies for cell therapy and specific disorder modeling [44].

### 4.1. iPSCs for Regenerative Cell Therapies Applied to Alzheimer’s and Parkinson’s Treatment

Implantation can range from raw iPSC populations to homogenous progenitor cell populations, to complex tissue-engineered constructs consisting of multiple cell species, for recovering regions damaged by ND. A number of studies demonstrated successful implantation of raw iPSCs into Alzheimer’s animal disease models. One such study showed that, after injecting iPSCs into 5XFAD Alzheimer’s mice, the iPSCs differentiated into glial cells upon implantation. Particularly, microglia, oligodendrocytes, and astrocytes were generated from the injected cells in vivo. The amount of Aβ plaque deposition decreased, along with a decrease in the activity of beta and gamma secretases, and increases in oligodendrocyte-related gene expression in the iPSCs-treated mice (Table 1) [45]. Additionally, iPSC-treated mice performed better on cognitive tasks, as evaluated by effectiveness in completing a maze. It is worth noting that the iPSCs used in this study were derived using a novel method, rendering the stem cells safer for transplantation and getting them closer to clinical trials. Typically, inducing somatic cells to reprogram into undifferentiated pluripotent stem cells required the introduction of exogenous genetic material via a viral vector [31]. This would cause the overexpression of a particular set of transcription factors, coined Yamanaka factors, leading to dedifferentiation. However, introduction of these genes augments the tumorigenic potential of the obtained stem cells and their derived tissues. Hence, in a previous study, Cho and his coworkers showed a protein-based method for inducing pluripotency without genetic modification, increasing safety, as compared to previous methods. It was performed via a streptolysin-mediated permeabilization protocol of proteins extracted from embryonic stem cells, which did not require long-term exposure of target cells to exogenous materials [46].

A number of studies have shown the survivability of autologous iPSC-derived dopaminergic neurons or progenitors in vivo, which was achieved by transplantation into primate or murine PD models [47,48,49] (Table 1). Implantation of iPSC-derived dopaminergic neurons, which have been differentiated in culture, into the putamen of Parkinsonian cygnus monkeys resulted in reinnervation via the implanted neurons, and improvements in motor function [48]. In another study, similarly, five Parkinsonian rhesus monkeys received autologous iPSC-derived dopaminergic neuron implants, and motor improvements were compared with those of another five Parkinsonian rhesus monkeys that received allogeneic transplantation. Midbrain dopaminergic neural progenitor cells were implanted into the four basal ganglia 1–3 years after induction of Parkinson’s via MPTP–HCL. Monkeys receiving autologous transplantation showed improvements in motor function as well as alleviation of mood disorder symptoms 24 months after surgery [47]. However, a unilateral model was used, so improvements may have been augmented by the healthy side [47], and no immunosuppression was used for the group receiving an allogeneic transplant. As improvements were correlated with the number of surviving dopaminergic neurons, suppression of the immune response to foreign dopaminergic neurons may have otherwise decreased their elimination, and allowed further alleviation of symptoms in monkeys receiving allogeneic transplants [47]. Another study by the Song research group generated clinical-grade midbrain dopaminergic neural progenitors from an iPSC cell line. Implantation of 100,000–300,000 of these cells into the striatum of immunodeficient Parkinson-induced mice yielded significant recovery in motor function after 14 weeks, which was sustained after at least 52 weeks. To bring these cells closer to clinical grade, they were produced under good manufacturing practice (GMP) using a specific protocol with the following key points: an episomal vector, along with selected miRNA, improved reprogramming efficiency of fibroblasts to iPSCs. A “spotting” technique was implemented, where cultures were split into smaller “spots”, yielding higher cell viability during growth and differentiation. Preimplantation, whole genome sequencing, karyotyping, and quantitative real-time polymerase chain reaction (qRT-PCR) were performed to ensure that selected iPSCs did not carry any known tumorigenic mutations nor integration of the episomal plasmid. The progenitors were also treated with quercetin to eliminate any leftover undifferentiated stem cells to avoid the risk of tumorigenesis upon implantation [49].

**Table 1:** Examples of preclinical trials using iPSC-derived cells for AD and PD disease treatment.

Source of iPSCs	Neurodegenerative Disease Treated	Model	Type of Cells	Number	Route of Delivery	Outcome	Reference
Autologous, mouse skin fibroblasts	AD	in vivo: 5XFAD mice	iPSCs	100,000	Injection into subiculum	Decrease in Aβ plaque deposition and beta/gamma-secretase activity	[45]
Autologous, skin fibroblasts	PD	in vivo: Parkinsonian cynomolgus monkeys	Dopaminergic neurons	10–40 million	Injection into four sites of post- commissural putamen	Improvements in motor function and reinnervation by implanted neurons	[48]
Autologous and allogeneic, skin fibroblasts	PD	in vivo: Parkinsonian rhesus monkeys	Dopaminergic neurons	5.5–22 million	Injection into basal ganglia	Improvements in motor function consistent with reinnervation by implanted neurons seen in autologous transplant group	[47]
Human dermal fibroblast lines	PD	in vivo: immunodeficient 6-OHDA Parkinsonian mice	Dopaminergic neuron progenitors	100,000–300,000	Injection into striatum	Recovery of rotation behavior, improvements on corridor, cylinder, stepping tests	[49]

The Schweitzer research group at Massachusetts General Hospital implanted human autologous iPSC-derived neural progenitor cells into a human subject. In preparation, a study was conducted to test the immunogenicity of iPSC-derived dopamine neural progenitor cells. They implanted midbrain dopaminergic progenitor cells generated from patient-derived iPSCs, as well as those from a different human iPSC line, into immune-deficient mice, K1 humanized mice, and mice with immune cells taken from the same patient (K1 mice had human immune cells but not from the patient). Both implant types survived in the immune-deficient mice, but were destroyed by the immune responses in K1 humanized mice, and only the patient-derived dopaminergic cells survived in the mice with the patient’s immune cells. The patient then received a graft of these dopaminergic progenitor cells to the putamen of both hemispheres at around 4 million cells per hemisphere. Throughout a period of 24 months following the initial operation, the patient showed increases of dopamine uptake by cells surrounding the implant site as evaluated by Flourodopa F18 (F-DOPA) positron emission tomography (PET). This was accompanied by improvements in Parkinsonian symptoms as evaluated by the Movement Disorder Society’s prescribed Unified Parkinson’s Disease Rating Scale (MDS–UPDRS), the Parkinson’s disease Questionnaire (PDQ-39), and the patient-recorded duration of “off times” [50]. Despite the very small sample size of one person, unknown long-term effects, and change in medication dosage being a possible interfering factor, the study showed barely significant but promising results [50]. While previous studies showed alleviated Parkinsonism in animal disease models upon implantation of iPSC-derived dopaminergic neuron progenitors [47,49], this was the first study that implanted the dopaminergic progenitor cells into a human subject.

So far, iPSC-derived products for PD and AD have not undergone many clinical trials. Apart from this study, the only example of iPSC-derived implantation into humans involves an ongoing clinical trial in Japan, for the treatment of PD, which was documented in a review by Ford and colleagues [51]. In Australia and China, hESC-derived cells are also undergoing clinical trials for treatment of PD [51]. Due to the aforementioned similarity between iPSCs and ESCs, it can be expected that iPSC-derived cells will also make their debut in clinical trials in the near future.

### 4.2. Using iPSC-Derived Organoids to Model Pathophysiology of Neurodegenerative Diseases

In addition to implantation for cell therapy, iPSCs can be used for the production of in vitro models of the afflicted region of the brain in ND patients. Since the donor’s iPSCs carry the same genotype as the donor, this is especially useful for understanding how genetic variations affect pathology, and can prove especially beneficial for personalized drug discovery aimed towards specific genetic variants. The pathophysiology of Alzheimer’s and Parkinson’s is affected by genetic variations from person to person, which plays a role in drug responsiveness. For example, it is known that the effectiveness of L-DOPA in treating Parkinson’s varies depending on genotype [52,53], and it was even found that donepezil, a widely prescribed drug for AD, worsens Alzheimer’s symptoms in patients with a certain genotype [54]. Hence, in vitro models encompassing the pharmacogenetic polymorphisms across the population may prove useful in developing personalized medication regimens. Disease models can greatly help in the quest to better understand the pathological progression of Parkinson’s and Alzheimer’s and to test drug candidates for safety and efficacy. For this purpose, neurodegenerative diseases can be induced in animals. However, animal models often do not recapitulate human in vivo pathophysiology accurately. Indeed, some drugs that have been deemed safe and effective in animal trials were found to show very different results in humans. Hence, tissues or organoids derived from human iPSCs, such as a “brain on a chip”, show great promise in closely recapitulating the human in vivo pathophysiology and in aiding in drug discovery. As previously mentioned, it is crucial to observe not only neurons but also glial cells, and the interplay of both cell species, in the progression of ND.

Yet another key component involves vascular cells, specifically at the blood–brain barrier (BBB). In vascular neurodegenerative disease, cytotoxicity occurs due to the breakdown of neurovascular units, which consist of a combination of vascular endothelial cells, pericytes, neurons, and neuroglia making up the BBB [55]. A study by Jagadeesan and his coworkers exemplified the modeling of the BBB on a “brain-on-a-chip” device. This was performed by seeding two adjacent channels, separated by a semipermeable membrane, with iPSC-derived cells. One channel representing the neural side was seeded using “EZ spheres”, which are clusters of neural progenitor cells. Putting them through a differentiation protocol yielded a coculture of mature neurons, neural progenitors, and astrocytes (a subpopulation of neuroglia). The second channel was seeded with brain microvascular endothelial cells (BMECs), which adhered to extracellular matrix (ECM) proteins that were chemically attached to the polydimethylsiloxane (PDMS) semipermeable membrane between the two channels. The proteins were optimally selected for these specific cell types, a requirement for proper cell adhesion and functional development. The final cell populations were assessed via immunocytochemical analysis, and the permeability of the interface between the two channels was determined. Vascular endothelial cells formed tight junctions with each other, as is seen in the natural BBB, but this only occurred in the presence of the neural cell populations, highlighting the importance of cell–cell signaling for proper BBB formation. The final construct allowed perfusion of the “blood” channel that is lined with the microvascular endothelial cells for the purposes of testing the permeability of various drugs across the BBB [56]. Since patient-specific iPSCs are used, the exact response of a patient’s neurovascular system on a drug can be ascertained. The modular nature of this “organ-on-a-chip” technology, used in conjunction with patient-specific iPSCs, leaves the system open to improvements towards iPSC differentiation and inclusion of additional cellular subpopulations, such as pericytes [56]. Prospectively, cells taken from an AD or PD patient can be used to accurately model in vivo pathogenesis.

The SNCA gene encodes the alpha-synuclein protein. It is strongly correlated to the onset of familial PD. Studies have demonstrated that a triplication of the SNCA gene causes an increase in the buildup of alpha-synuclein deposits in iPSC-derived neural progenitor cells, which inhibits their maturation [57]. To build on this, Mohamed and his coworkers pioneered the use of 3D human midbrain organoids (hMOs) derived from a patient’s iPSCs for modeling SNCA triplication in order to investigate the effect of SNCA triplication on PD pathology. The organoids with triplication exhibited the expected Parkinson’s pathophysiology, with a lower number of dopaminergic neurons, and a decrease in size after 100 days, compared to an isogenic control. This method paves the way for more accurate disease modeling, as 3D midbrain organoids can survive for longer, allowing the deduction of age-dependent changes. Furthermore, the 3D organoids contain many types of cells, allowing closer mimicry of the cell–cell interactions in vivo [58]. The Raja research group developed scaffold-free, 3D self-assembling neural organoids that demonstrate the hallmark signs of Alzheimer’s: tauopathy and Aβ accumulation. They also demonstrated “in vitro aging” of the organoids and their platform was highly responsive to drugs, allowing for observation of phenotypic changes relating to the application of beta or gamma secretase inhibitors [5]. Using this 3D model, which more closely resembles actual Parkinsonian progression, would improve the discovery and testing of drugs moving towards preclinical drug validation. Nevertheless, it was noted that the exact correlation between the “in vitro aging” of the organoids and the maturation of an actual human brain is yet to be determined [5], which is critical for further research. A further study by the Mohamed research group developed a method aimed at addressing issues with the scale up of organoid production. They proposed the packaging of iPSC-derived brain organoids on 948-well, microfabricated disks of Matrigel. This method ameliorated the efficiency of hMO production on a large scale, yielding lower cost and batch-to-batch variability. It does not require automation, making it more accessible to labs with only basic equipment [59].

With the production of neural constructs, the major issue of vascularization remains. A construct with cells further than 200–400 microns from surface contact with the culture medium must have an adequate vascular network to supply nutrients, remove waste, and transport cell signaling molecules, due to the diffusion limits. In a recent study, researchers coated whole-brain organoids derived from a patient’s iPSCs in a layer of Matrigel seeded with endothelial cells (ECs), which were derived from the same patient’s iPSCs. This resulted in angiogenesis of the organoid from the outside in. Implantation of the construct into a mouse model resulted in deeper penetration of the vascular network by the human ECs when compared to just in vitro vascularization. This is promising for the future of larger neural implants and organoids, which must overcome the lack of blood vessel in vitro [60]. Nevertheless, more work is needed to determine the exact cell signal cascade that augmented this vascularization process in vivo. Its translation would allow growth of larger organoids in vitro using a specifically defined medium conducive to angiogenesis.

### 4.3. Bioprinting for Regeneration and In Vitro Neurodegenerative Disease Modeling

#### 4.3.1. Utility of Bioprinter for Making Tissue Constructs

A tissue-engineered construct can be implanted to replace damaged tissue or used to accurately model the disease environment, lending itself to drug discovery and testing [61]. To engineer a tissue construct, three components are typically required: cells, dissolved signaling molecules, and a scaffold [62]. Patient-specific iPSCs are ideal candidates for building a tissue construct for pathological modelling or implantation. Dissolved signaling molecules around the cells are crucial for guiding cell behavior, and therefore the response of implanted cells to dissolved factors in situ should be carefully considered. An implanted construct could be supplemented with additional signaling molecules to guide cell fate in vivo. Cells also respond to physical and mechanical cues in their surroundings, naturally conferred by the ECM in vivo. A scaffold takes the place of the ECM in the context of an engineered construct; its mechanical properties should correspond to those specifically required by the desired cell type. It has also been shown that development of some cell types, namely neurons, benefits from electrical stimulation [63], which should undoubtedly be taken on board when developing a tissue-engineered neural construct. Bioprinting is an exciting new field that shows great promise in manufacturing such constructs by spatially depositing the scaffold material as well as cells, all the while allowing for controlled encapsulation of soluble cell signaling ligands. Using a bioprinter, parameters including the chemical and physical properties of the bioink, and the 3D structure of the construct can be adjusted.

Conventionally, a hydrogel laden with cells, such as iPSCs or differentiated cells, can be deposited with precision, forming a 3D shape. It is then hardened via crosslinking to consolidate the 3D geometry and provide mechanical support. This may be undertaken during or post-printing, via introduction of metallic ions, photopolymerization, or thermogelation [64]. Porosity, mechanical properties, cell adhesion, and crosslinking technique are features that must be balanced in the selection of the optimal hydrogel bioink [55]. Crosslinking has the potential to damage the cells within the bioink, as it exerts stresses on them in the form of mechanical shear, temperature variation, ionic changes, and laser radiation. This should especially be considered when using iPSCs, which are extremely sensitive to mechanical stresses [63]. A higher stiffness usually yields higher print resolution [55], but requires more extrusion force to print, putting cells at higher risk of damage. Notably, suspension printing offers a clever work around, whereby a less viscous bioink, which does not rely on high shear thinning nor on fast crosslinking, can be used without compromising on print resolution [55]. The lower viscosity bioink is extruded into a bath of self-healing gel, which provides support while printed material ink is crosslinked [55]. Mechanical properties such as stiffness influence cell behavior, as cells physically anchor themselves to the extracellular scaffold, and pick up on mechanical feedback via mechanotransduction, triggering an intracellular biochemical signaling cascade. Typically, cell adhesion to a surface is required for cell differentiation and function, which requires specific proteins optimized for that cell type [56]. Appropriate selection of porosity should permit adequate perfusion throughout the construct, without sacrificing structural integrity.

#### 4.3.2. Bioprinting iPSC-Derived Brain Cells

For the first time, Gu and his coworkers printed iPSCs that were encapsulated in a porous scaffold consisting of alginate, carboxymethyl chitosan, and agarose [65]. After extrusion printing, the constructs were crosslinked using calcium ions to consolidate the 3D architecture and to confer the desired mechanical properties for iPSC survival. The iPSCs proliferated successfully on the pore walls within the constructs for 9 days, after which their growth underwent contact inhibition, as expected for mammalian cells in vivo. Next, the constructs were exposed to specific neuronal differentiation media for the formation of functional neurons and supporting neuroglial cells. Both GABAergic and serotonergic neurons were produced in addition to a diverse number of glial cells such as oligodendrocytes and astrocytes, as evaluated by immunophenotyping for cell type-specific markers. Neuronal cells within the construct exhibited neurite outgrowth and migration, as well as synaptic activity as demonstrated by calcium imaging [65]. These constructs were the first of their kind, offering a method to produce tunable 3D structures that support iPSC viability and differentiation. This study exhibited that their constructs supported conditions for heterogeneous neural and glial cell populations, which could see applications in regenerative medicine: a similar construct could be implanted into Parkinson’s and Alzheimer’s patients for the purpose of recuperating tissue lost to neurodegeneration. This would contain autologous iPSC-derived cells and the 3D architecture could be personalized to the individual. In situ, the iPSCs within the construct would differentiate due to native signals secreted from the in vivo environment or supplementary growth factors. However, this technique has yet to undergo optimization for animal trials and clinical use. Before then, it would be beneficial to assess whether the cell proportions truly mimic those in vivo, and refine methods for a robust approach applicable to different patients.

It has been known that the neural networks to be repaired are complex and specifically wired. Researchers optimized the bioink composition for iPSC proliferation and differentiation, and displayed successful neuronal function within their printed constructs [65]. However, the arrangement of the neural cells was not controlled. Therefore, it would be beneficial for further studies to look into directing specific placement and neurite outgrowth to mimic native connectivity. The Shimba research group proposed using 3D-printed PDMS scaffolds to guide neurite outgrowth in order to form a specific network between neural cells. Multielectrode arrays (MEAs) would be used to verify the connectivity between neurons [66]. The challenge, however, remains in ascertaining the exact large-scale neural pattern or the “connectome” of the tissue being replaced [67]. It is expected that a customized implant could be manufactured to replace the neural networks that have been degraded due to neurodegenerative progression [66].

Bioprinting neural cell-laden constructs can be applied to personalized ND modeling in vitro, for the purpose of pharmaceutical drug discovery and perhaps even bedside drug testing. The Yi research group aimed to ascertain the best combination of drugs as well as to predict the efficacy of chemotherapy for the specific patient using a bioprinting approach [68]. This study engineered brain organoids using patient-derived glioblastoma cells and vascular ECs that were seeded in a decellularized ECM bioink. The bioprinted construct captured the cell–cell and cell–environment interactions, which are key to modeling the overall tissue behavior in vivo and determining patient-specific differences. It is predicted that, once guided neurite outgrowth becomes feasible through 3D bioprinting, modeling specific neural networks could prove extremely useful for studying the specific effects of ND pathogenesis as well as for in vitro pharmacology aimed towards drug discovery [69].

#### 4.3.3. Bioprinting iPSC-Derived Constructs for Regenerative Cell Therapy

In the context of neural tissue, where the precise cytoarchitecture is paramount, bioprinting might aid in the regenerative treatment of ND. A 3D-printed construct can more closely mimic the original in vivo cytoarchitecture [65] and, hence, may integrate more effectively in vivo. As a prime example of this, the Joung research group bioprinted a neural construct mimicking a section of a rat spinal cord. The iPSC-derived spinal neural progenitor cells and oligodendrocyte progenitor cells were cultured and then coprinted within microchannels of a Matrigel scaffold. Their platform, intended for spinal cord regeneration, demonstrated a successful technique for depositing multicellular cell-laden bioinks into precise biomimetic shapes, which resulted in the effective guidance of differentiation and neurite outgrowth into the scaffold [70]. However, further research is required to determine whether this approach can be applied for precise tissue regeneration of brain regions damaged by neurodegeneration.

Bioprinting may also lend itself to printing neurovascular units, which model the complex in vivo interactions between vascular and neural cells in vitro. Previous studies successfully recapitulated the human BBB in a human brain organoid that was implanted into rodents for simulating drug permeability across the BBB. A major issue with this technique, however, is the lack of reproducibility, owing to the variability of vascularization and organoid morphology [71]. Bioprinting has the potential to remedy this problem of brain organoid manufacture in the future, as it allows the exact placement of both cells and their guiding scaffolds. A number of studies have shown promising results in the field of printing 3D neural constructs (Table 2). However, studies concerning its application in vivo are currently lacking.

## 5. Limitations and Future Directions

Currently, a number of limitations are standing in the way of iPSC-derived regenerative cell therapy: (1) A characteristic of iPSCs is their neoplasticity; they readily form teratomas in vivo. Upon implantation they may differentiate into an unforeseen lineage or proliferate uncontrollably, causing issues in the host. Hence, possible contamination by undifferentiated cells remains a prominent concern upon implanting iPSC-derived cells. (2) Another limitation exists in the genetic aberrations [49,76,77,78] inherited from the donor tissue, and those accumulated during proliferation in vitro. This increases the risk of tumorigenicity for the implanted tissue. Indeed, preliminary clinical trials using iPSC derived cell therapy were halted due to this issue [49,79]. (3) The familial form of AD is difficult to treat with autologous stem cell-derived cells, as they will contain the same disease-causing genes as the patient. Implanting them without some modification may prove futile.

To address the first two limitations, the aforementioned studies by Song et al. and Schweitzer et al. used a contemporary protocol in which they performed whole-exome sequencing (WES) and genome sequencing (WGS) to check for known tumorigenic mutations prior to implantation, and treated the midbrain dopaminergic progenitors (mDAPs) with quercetin. Schweitzer et al. subsequently examined undifferentiated cell-marker expression using flow cytometry and qRT-PCR to ensure the absence of iPSC contamination [50]. Although Schweitzer et al. implanted iPSC-derived cells for treatment of PD, larger-scale human trials would be needed to evaluate the response to regenerative therapy based on genetic diversity [50]. Song et al. dedicated their focus to ensuring clinical purity and scalability by using the WGS and quercetin treatment for safety by integrating improvements in iPSC differentiation technique. Importantly, they showed that their protocol could be scaled up under good manufacturing practices (GMP) [49]. These sustained efforts to develop clinical-grade iPSC products and to define gold standards would lead to large-scale clinical trials involving iPSC-derived regenerative therapy for ND in the near future.

Likewise, there are limitations with iPSC-based in vitro modeling. A majority of induced disease models are based on familial AD since they are generated by genetic engineering. However, the majority of AD and PD cases in humans are sporadic [10,80], meaning they are caused by accumulated change during the lifetime rather than by inherited genes. This renders the translation accuracy between the model and actual pathogenesis questionable, although there are undoubtedly similarities between familial and sporadic cases [3] such as the increased levels of SNCA expression, which is the pathological hallmark of the organoids generated by Mohamed and colleagues [58].

Another limitation of the current technologies using iPSC for ND treatment is that implantation of iPSCs for treatment of ND was carried out by injecting a homogeneous suspension culture of cells, without a 3D structure. The formation of an expected 3D architecture relied on subsequent self-assembly and integration in situ after implantation. Yet to be implemented, the integration of the protocols developed by Joung et al. with the proposed approach of Shimba and colleagues may yield a revolutionary way to precisely regenerate specific 3D neural networks lost due to Parkinson’s and Alzheimer’s diseases [66]. As previously mentioned, this is contingent on the accurate deduction of patient-specific connectomes.

More research is needed on modeling in vitro organoids with a more diverse population of cell types to better approximate in vivo ecosystems. For example, the inclusion of circulating immune cells, which undoubtedly play a role in the neurodegenerative environment [81], could be considered for further studies. Bioprinting iPSC-derived constructs offers significant advantages in quality, precision, and reproducibility in the context of implantation and in vitro modeling. Nonetheless, the in vivo efficacy of 3D-printed neural constructs for ND remains incomplete. Future directions will entail the optimization of biomaterials to develop optimal bioinks for guiding neural cell differentiation [63]. Ideally, the scaffold of a 3D-printed implant should gradually be replaced by native ECM. This prospect relies on advances in biomaterial science geared towards matching the biodegradation rate of bioinks to ECM deposition rate [63]. Taken together, as this review has outlined, fundamental advantages offered by iPSCs, especially coupled with the exciting new prospects of 3D bioprinting and its endless modalities, show promise in understanding and treating Alzheimer’s and Parkinson’s through in vitro modeling and personalized regenerative cell therapy in the future.

## Figures and Tables

**Table 2:** Examples of 3D-bioprinted neural constructs seeded with iPSC-derived cells.

Strategy	Scaffold Materials	Seeded Cell Type	Outcome	Reference
Extrusion-printed stem cells within scaffold, which were then differentiated into different lineages depending on culture medium.	Alginate, Carboxymethyl Chitosan, Agarose	iPSCs	iPSCs within the bioink differentiated into GABAergic and serotonergic neurons, neuroglia; in vitro functionality shown by neuron migration.	[65]
Extrusion-printed artificial spinal cord consisting of multiple cell types in microchannels within scaffold.	Alginate, Methylcellulose	Spinal neural progenitor cells and oligodendrocyte progenitor cells	Guided differentiation and neurite outgrowth in vitro, neuron functionality shown by calcium imaging.	[70]
Printed cell aggregates embedded within microchannels of a novel scaffold material, tested cell viability and morphology.	Fibrin-based bioink	Neural progenitor cells	Cells within the scaffold displayed TUJ1 neuronal marker and neurite outgrowth after 41 days of culture.	[72]
Printed dome-shaped neural tissue structure consisting of neural progenitor cells and drug-eluting microspheres	Fibrin-based bioink with guggulsterone-eluting microspheres	Neural progenitor cells	Drug elution induced differentiation of progenitors into dopaminergic neurons, oligodendrocyte progenitors, and other glial cells after 30 days.	[73]
Printed neural progenitor cells embedded in bioink along with drug-loaded microspheres.	Fibrin-based bioink with retinoic acid, polycaprolactone, purmorphamine-eluting microspheres	Neural progenitor cells	Progenitors differentiated into GABAergic and cholinergic neurons, astrocytes, and oligodendrocytes. Neurons responded to neurotransmitter after 30–45 days in culture.	[74]
Extrusion printed cells within novel bioink blend, tested electrophysiological behavior.	Blends of Alginate, Gellan gum, and Laminin	Neural progenitor cells	Progenitors differentiated into dopaminergic neurons and astrocytes after 21 days; neurons were electrically active, showed migration and outgrowth.	[75]

## Data Availability

Not applicable.
